# A Heat-Inactivated Two-Strain *Lacticaseibacillus paracasei* Fermented Milk as a Postbiotic for Functional Constipation: A Randomized, Double-Blind, Placebo-Controlled Trial

**DOI:** 10.3390/nu18132114

**Published:** 2026-06-28

**Authors:** Xinyi Li, Hanglian Lan, Yiran Guan, Langrun Wang, Wen Zhao, Jian He, Zhi Zhao, Meina Li, Qixu Han, Yifan Gong, Xinxin Yan, Ziwen Li, Jie Guo, Ran Wang, Jingjing He

**Affiliations:** 1 Key Laboratory of Precision Nutrition and Food Quality, Department of Nutrition and Health, China Agricultural University, Beijing 100193, China; sy20243313908@cau.edu.cn (X.L.); b20253311579@cau.edu.cn (Y.G.); b20243311365@cau.edu.cn (L.W.); zxz0604@yeah.net (Z.Z.); 15122295019@163.com (M.L.); hqx@cau.edu.cn (Q.H.); 1073323120744@st.gsau.edu.cn (Y.G.); yxx2025331001@cau.edu.cn (X.Y.); guojie@cau.edu.cn (J.G.); 2 National Technology Innovation Center for Dairy, Hohhot 010100, China; mindylan678@gmail.com (H.L.); 13614317400@163.com (W.Z.); hejian@nctid.cn (J.H.); 3 School of Food and Health, Beijing Technology and Business University, Beijing 100048, China; rrjlydia@163.com; 4 Research Center for Probiotics, China Agricultural University, Beijing 100190, China

**Keywords:** functional constipation, heat-inactivated *Lacticaseibacillus paracasei*, randomized controlled trial, vasoactive intestinal peptide, acetylcholine

## Abstract

Background/Objectives: Functional constipation (FC) commonly affects middle-aged and older adults, but current pharmacological treatments have limitations. Postbiotics may offer safety advantages, but clinical evidence is limited. This randomized controlled trial evaluated the efficacy and safety of a heat-inactivated two-strain *Lacticaseibacillus paracasei* fermented milk in adults with FC. Methods: One hundred adults aged 45–75 years with Rome IV-defined FC received the fermented milk or placebo for 4 weeks. The primary outcome was the change in weekly spontaneous bowel movement (SBM) frequency from baseline to week 4. Secondary outcomes included complete spontaneous bowel movement (CSBM) frequency, whole-gut transit time (WGTT), constipation symptom scores, quality of life, serum biomarkers, and adverse events. Primary analysis was per-protocol (*n* = 96); intention-to-treat analysis was applied to primary and key secondary outcomes. Results: Dropout was 4% (*n* = 4, 2 per group), and adherence was >80% in both groups. The intervention showed no significant benefit over placebo for the primary outcome or for most secondary clinical outcomes. Although both groups improved within-group, no significant between-group differences were observed at week 4 for changes in SBM (MD = −0.14, 95% CI: −0.85, 0.57; *p* = 0.683), CSBM (MD = 0.27, 95% CI: −0.61, 1.15; *p* = 0.543), or WGTT (MD = −1.55 h, 95% CI: −7.65, 4.55; *p* = 0.614). Symptom and quality-of-life scores also did not differ between groups. Exploratory biomarker analyses showed significantly greater increases in serum VIP and ACh in the intervention group (VIP: MD = 105.23 ng/L, *p* < 0.001; ACh: MD = 42.95 ng/L, *p* = 0.035). No adverse events were reported. Conclusions: Four weeks of this postbiotic was safe but did not significantly improve bowel function or symptoms in the overall FC population. The increases in serum VIP and ACh suggest engagement of neurotransmitter-related pathways; however, these exploratory findings do not imply causation or clinical efficacy and warrant confirmation in longer-duration trials (Clinical Trial Registry: ChiCTR2500111771).

## 1. Introduction

Functional constipation (FC) is a common functional gastrointestinal disorder [1], with a global pooled prevalence of approximately 10.1% [2]. It is particularly relevant among middle-aged and older adults, with a reported prevalence of 17.60% among Chinese individuals aged 65 years or older [3]. FC not only impairs quality of life but also increases healthcare burden [4,5]. Current pharmacological treatments are limited by interindividual variability, poor long-term adherence, and adverse effects [6,7]. Therefore, safe and stable non-pharmacological strategies for long-term management are needed.

Gut microbiota dysbiosis is implicated in FC pathophysiology, with reduced abundances of beneficial taxa such as *Bifidobacterium* and *Lactobacillus* [8,9], which may contribute to impaired defecatory function through microbiota–gut–brain axis signaling [10,11,12]. Although some live probiotics have shown clinical benefits [13,14,15], the available evidence in adults with chronic constipation or diagnosed FC remains variable. From a biological and product-development perspective, probiotic effects may be limited by strain specificity, survival in the gastrointestinal tract, and stability during manufacturing and storage [16,17,18,19]. From a clinical perspective, treatment effects may also differ according to dose, intervention duration, delivery matrix, baseline symptom severity, and outcome definitions [20,21]. Moreover, placebo-controlled trials in Rome-defined FC populations have not uniformly shown superiority over placebo [22]. More recently, an 8-week trial of *Bifidobacterium lactis* HN019 in adults with FC reported improvements in CSBM frequency in both groups, but HN019 did not outperform placebo [23]. These findings indicate that microbiota-targeted interventions should be evaluated in a strain-, formulation-, and population-specific manner, rather than assuming that benefits observed with one product can be generalized to another.

Compared with live probiotics, postbiotics—preparations of inanimate microorganisms and/or their components that confer a health benefit on the host—have been proposed to offer advantages in safety, stability, and dose standardization [24,25]. Heat-inactivated postbiotic preparations have been reported to improve bowel movement frequency and defecation-related outcomes in healthy or constipation-prone individuals [26,27]. These effects may be related to retained microbial structures and fermentation metabolites that modulate intestinal water secretion, mucosal barrier function, inflammatory responses, and neurotransmitter-related signaling [28,29,30,31,32]. However, most available studies on heat-inactivated postbiotic preparations have been conducted in healthy, constipation-prone, or heterogeneous constipation populations rather than Rome-defined FC populations, and differences in strains, inactivation methods, formulations, and food matrices limit direct comparison across studies. Therefore, evidence for the efficacy of heat-inactivated postbiotic preparations in individuals with diagnosed FC remains limited, and well-controlled trials are needed to determine whether such products provide clinically meaningful benefits in FC populations.

*Lacticaseibacillus paracasei* K56, isolated from the gastrointestinal tract of healthy Chinese infants, has shown metabolic and gut microbiota-modulating effects [33,34] and has been reported to promote defecation-related outcomes in animal models [35]. However, evidence on its heat-inactivated form in FC is lacking. Given that FC involves multiple pathophysiological processes, a single-strain postbiotic may not fully address this complexity, whereas a two-strain preparation may broaden the spectrum of bioactive components. We therefore developed a heat-inactivated fermented milk combining K56 with a complementary *L. paracasei* strain (G078), selected for its compatibility with K56 during milk co-fermentation and its contribution to the fermentation properties of the final product. Whether this two-strain postbiotic preparation provides clinically meaningful benefits in FC populations has not been established. This intervention study therefore evaluated its effects on clinical outcomes, serum neurotransmitter-related biomarkers, and inflammatory cytokines in middle-aged and older adults with FC.

## 2. Materials and Methods

### 2.1. Study Design

This randomized, double-blind, placebo-controlled, parallel-group clinical trial was approved by the Human Research Ethics Committee of China Agricultural University on September 1, 2024 (Approval No.: CAUHR-20241001) and registered with the Chinese Clinical Trial Registry on November 5, 2025 (registration No. ChiCTR2500111771). The study was conducted in Beijing, China, in accordance with the Declaration of Helsinki [36] and applicable Chinese laws and regulations governing clinical research.

**Figure 1:**
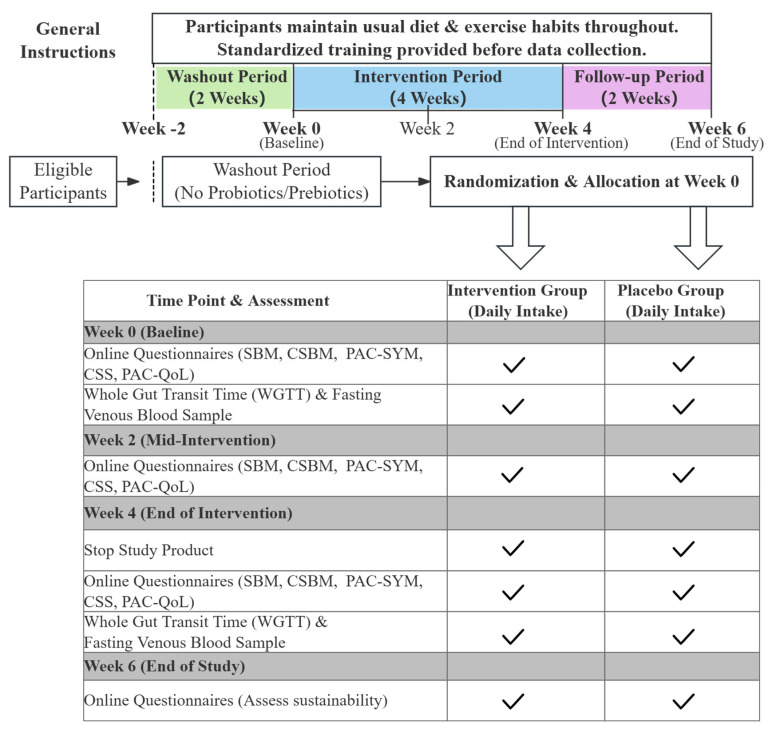
Study design and assessment schedule. Colors indicate study phases (washout, intervention, and follow-up). Arrows represent the chronological progression of the study timeline and participant flow, including randomization and group allocation. Check marks (√) indicate time points at which specific assessments or procedures were performed. CSBM, complete spontaneous bowel movement; CSS, Constipation Scoring System; PAC-SYM, Patient Assessment of Constipation Symptoms; PAC-QoL, Patient Assessment of Constipation Quality of Life; SBM, spontaneous bowel movement; WGTT, whole-gut transit time.

Eligible participants first entered a 2-week washout period, during which they were required to avoid probiotics, prebiotics, and fermented dairy products to minimize potential background dietary effects. After the washout period, participants were randomly assigned to the intervention group (receiving the heat-inactivated fermented milk) or placebo group, and received the assigned intervention for 4 weeks, followed by a 2-week post-intervention follow-up (Figure 1). Participants completed online questionnaires at baseline (week 0), week 2, week 4, and week 6 to assess constipation symptom severity and quality of life. In-person visits at weeks 0 and 4 included fasting venous blood collection and whole-gut transit time (WGTT) assessment. Throughout the trial, participants were instructed to maintain their usual diet and physical activity habits. All participants received standardized training on study procedures before data collection.

### 2.2. Participants and Eligibility Criteria

The study was conducted in Beijing, China, from November 2025 to March 2026. Participants were recruited through online advertisements and offline promotional materials. A total of 100 eligible participants provided written informed consent and were enrolled. Eligible participants were men and women aged 45–75 years who met the Rome IV diagnostic criteria for FC and had a body mass index (BMI) of 18.5–34.9 kg/m^2^. Key exclusion criteria were: (1) severe gastrointestinal diseases (e.g., gastric ulcer, duodenal ulcer, and ulcerative colitis), organic lesions (e.g., intestinal cancer), or chronic diseases such as type 1 diabetes or Parkinson’s disease; (2) use of H2-receptor antagonists, proton pump inhibitors, probiotics, or prebiotic preparations within 2 weeks before the trial; (3) use of prokinetic agents, opioids, antispasmodics, nonsteroidal anti-inflammatory drugs, antibiotics, or laxatives within 3 days before randomization; (4) excessive smoking (>20 cigarettes/day) or alcohol abuse (>500 mL of Chinese baijiu (distilled spirits) per week, ≥40% alcohol by volume); (5) pregnancy, lactation, or planned pregnancy within 6 months; (6) allergy to milk or soy; and (7) any condition judged by the principal investigator to make the participant unsuitable for the study. The full eligibility criteria are provided in the Supplementary Table S1.

### 2.3. Randomization and Blinding

Eligible participants were randomly assigned in a 1:1 ratio to the intervention group or placebo group using a dynamic randomization method (minimization). The allocation sequence was computer-generated by an independent statistician not involved in participant recruitment, intervention delivery, outcome assessment, or data analysis. Gender (self-reported as men or women), age (<64 years vs. ≥64 years, based on the baseline median), and baseline weekly spontaneous bowel movement (SBM) frequency (<3/week vs. ≥3/week) were included as allocation factors to ensure balance between groups.

The trial was double-blind. Participants and investigators remained blinded to group allocation throughout the trial. The intervention and placebo products were identical in appearance, packaging, and labeling to maintain blinding.

### 2.4. Intervention

The intervention product was a heat-inactivated fermented milk produced by co-fermentation using *L*. *paracasei* K56 and *L*. *paracasei* G078. Quality control confirmed that the co-fermented culture had a total viable count of approximately 1.5 × 10^9^ CFU/g before heat inactivation. In the final intervention product, each 100-mL bottle contained heat-inactivated bacterial cells equivalent to approximately 3.9 × 10^10^ CFU of K56 and G078 combined before heat inactivation.

The placebo was a nutritionally and sensorily matched milk-based matrix without the study strains (Supplementary Table S2). Both the intervention and placebo products, along with the study strains, were manufactured and supplied by Inner Mongolia Yili Industrial Group Co., Ltd. (Hohhot, Inner Mongolia, China). Participants consumed two 100-mL bottles daily, one after breakfast and one after dinner. At week 0, participants received the full 4-week supply of study products and were instructed to store them at room temperature. Compliance was assessed by counting returned bottles, and consumption of <80% of the assigned products was defined as a protocol deviation for the per-protocol analysis.

### 2.5. Outcome Assessments

#### 2.5.1. Primary Outcome

The primary outcome was the change in weekly SBM frequency from baseline to week 4. SBM was defined as a bowel movement occurring without the use of rescue medications (e.g., laxatives) or enemas [1]. Participants recorded bowel movements and related defecation information in a daily bowel movement diary, which was used to calculate weekly SBM frequency.

#### 2.5.2. Secondary Outcomes

##### Complete Spontaneous Bowel Movement

A complete spontaneous bowel movement (CSBM) was defined as a bowel movement occurring without rescue medications or enemas and accompanied by a sensation of complete evacuation [1]. Weekly CSBM frequency was calculated from daily bowel movement diaries.

##### WGTT

WGTT was defined as the time from ingestion of a marker to its first excretion in stool [4,37]. WGTT was measured at baseline and week 4 using the blue cake method. This method shows good agreement with the radiopaque marker method and is radiation-free, low-cost, and acceptable to participants [38]. Participants consumed a standardized blue cake immediately after their last bowel movement and recorded the exact time of cake ingestion and the first appearance of blue stool. WGTT was calculated as the time interval between ingestion and the first passage of blue stool. During the test period, participants avoided other blue- or purple-colored foods. The cake recipe is provided in the Supplementary Materials.

##### Constipation Symptom Severity and Quality of Life

Constipation symptom severity was assessed using the Patient Assessment of Constipation Symptoms (PAC-SYM; 12 items, 0–48) [39] and the Constipation Scoring System (CSS; 8 items, 0–30) [40,41]. Constipation-related quality of life was assessed using the Patient Assessment of Constipation Quality of Life (PAC-QoL; 28 items, 0–112) [42]. Higher scores indicate worse symptoms or greater impairment of life quality. Validated Chinese versions were used for all scales.

##### Serum Biomarker Assessment

Morning fasting venous blood samples were collected at baseline (week 0) and at the end of the intervention (week 4). Serum was separated, aliquoted, and stored at −80 °C until biochemical analysis. Serum 5-hydroxytryptamine (5-HT), vasoactive intestinal peptide (VIP), acetylcholine (ACh), interleukin-1β (IL-1β), interleukin-6 (IL-6), interleukin-10 (IL-10), tumor necrosis factor-α (TNF-α), and interferon-γ (IFN-γ) were measured using enzyme-linked immunosorbent assay (ELISA) kits (Shanghai Yuanju Biotechnology Co., Ltd., Shanghai, China). Serum nitric oxide (NO) concentrations were measured using an NO assay kit (Jiangsu Addison Biotechnology Co., Ltd., Yancheng, Jiangsu, China). All assays followed the manufacturers’ instructions, and all intra-assay and inter-assay coefficients of variation (CVs) were <10%.

### 2.6. Adverse Events

Adverse events were monitored at each visit using open-ended questioning. Causality and seriousness were adjudicated by the principal investigator.

### 2.7. Sample Size Calculation and Statistical Analysis

#### 2.7.1. Sample Size Calculation

The sample size was calculated based on the primary outcome—change in weekly SBM from baseline to week 4 (ΔSBM). Based on previous studies, the expected between-group difference in ΔSBM was 0.98 per week [43], with an assumed standard deviation of 1.6 [44]. With a two-sided α of 0.05 and 80% power, the minimum required sample size was 42 participants per group (PASS 15.0 software, NCSS, LLC, Kaysville, UT, USA). Assuming an approximately 20% dropout rate, the target sample size was increased to 50 participants per group, for a total of 100 participants.

#### 2.7.2. Statistical Analysis

Analyses were primarily performed using the per-protocol set (PPS), which included participants who completed the 4-week intervention, had compliance ≥ 80%, and had no major protocol deviations. Intention-to-treat (ITT) analysis was performed for the primary outcome (change in weekly SBM frequency from baseline to week 4) and for the key secondary outcomes (CSBM frequency and WGTT at week 4). Given the low dropout rate (4%), missing data in the ITT analysis were imputed using last observation carried forward (LOCF). Statistical analyses were conducted using SPSS version 26.0 (IBM Corp., Armonk, NY, USA) and GraphPad Prism version 8 (GraphPad Software, Inc., La Jolla, CA, USA). All tests were two-sided, and *p* < 0.05 was considered statistically significant.

Continuous variables with normal or approximately normal distributions were expressed as mean ± standard deviation (SD), whereas skewed variables were expressed as median and interquartile range (IQR). Categorical variables were expressed as frequencies and percentages.

The primary analytical variable was the change from baseline (Δ = value at each visit-baseline value). Within-group comparisons were performed using paired-samples t-tests or Wilcoxon signed-rank tests, depending on data distribution. Between-group comparisons used independent-samples t-tests or Mann–Whitney U tests. Categorical variables were analyzed using chi-square tests.

Exploratory analyses were performed according to baseline SBM frequency (<3 vs. ≥3 movements/week). Between-group differences in changes from baseline to week 4 were evaluated for SBM, CSBM, and WGTT within each subgroup. An exploratory responder analysis was also conducted. Responders were defined as participants who achieved an increase of at least one CSBM per week from baseline to week 4. Responder rates were calculated for the overall PP population and further described according to baseline SBM frequency. Between-group differences in responder rates were assessed using Fisher’s exact test. These analyses were considered supplementary and hypothesis-generating, with no adjustment for multiple comparisons.

## 3. Results

### 3.1. Characteristics of Participants

**Figure 2:**
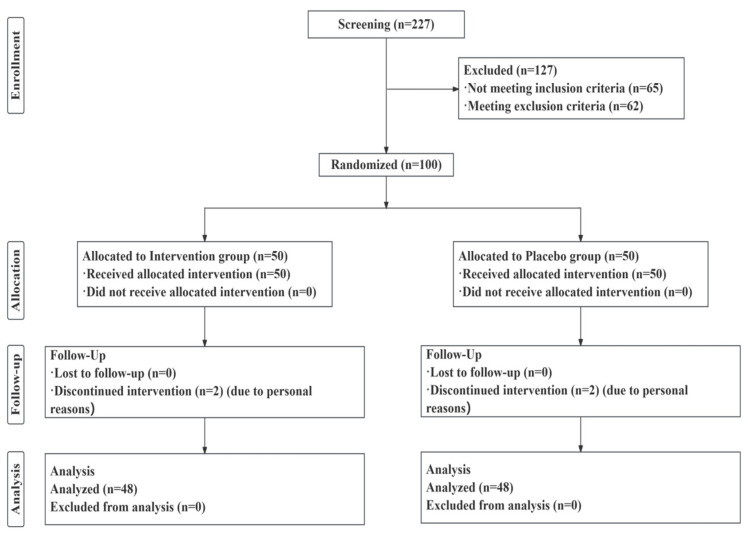
CONSORT flow diagram of participant enrollment, allocation, follow-up, and analysis.

A total of 227 participants were screened, and 100 eligible participants were randomly assigned to the intervention group (*n* = 50) or placebo group (*n* = 50). During the trial, four participants withdrew for personal reasons, leaving 96 participants who completed the study, all of whom had compliance ≥ 80% and were included in the PP analysis (Figure 2). Baseline characteristics, including age, gender, BMI, SBM frequency, CSBM frequency, and WGTT, were comparable between the two groups in the ITT population (all *p* > 0.05; Table 1).

### 3.2. Clinical Outcomes

#### 3.2.1. Bowel Movement Frequency and WGTT

**Figure 3:**
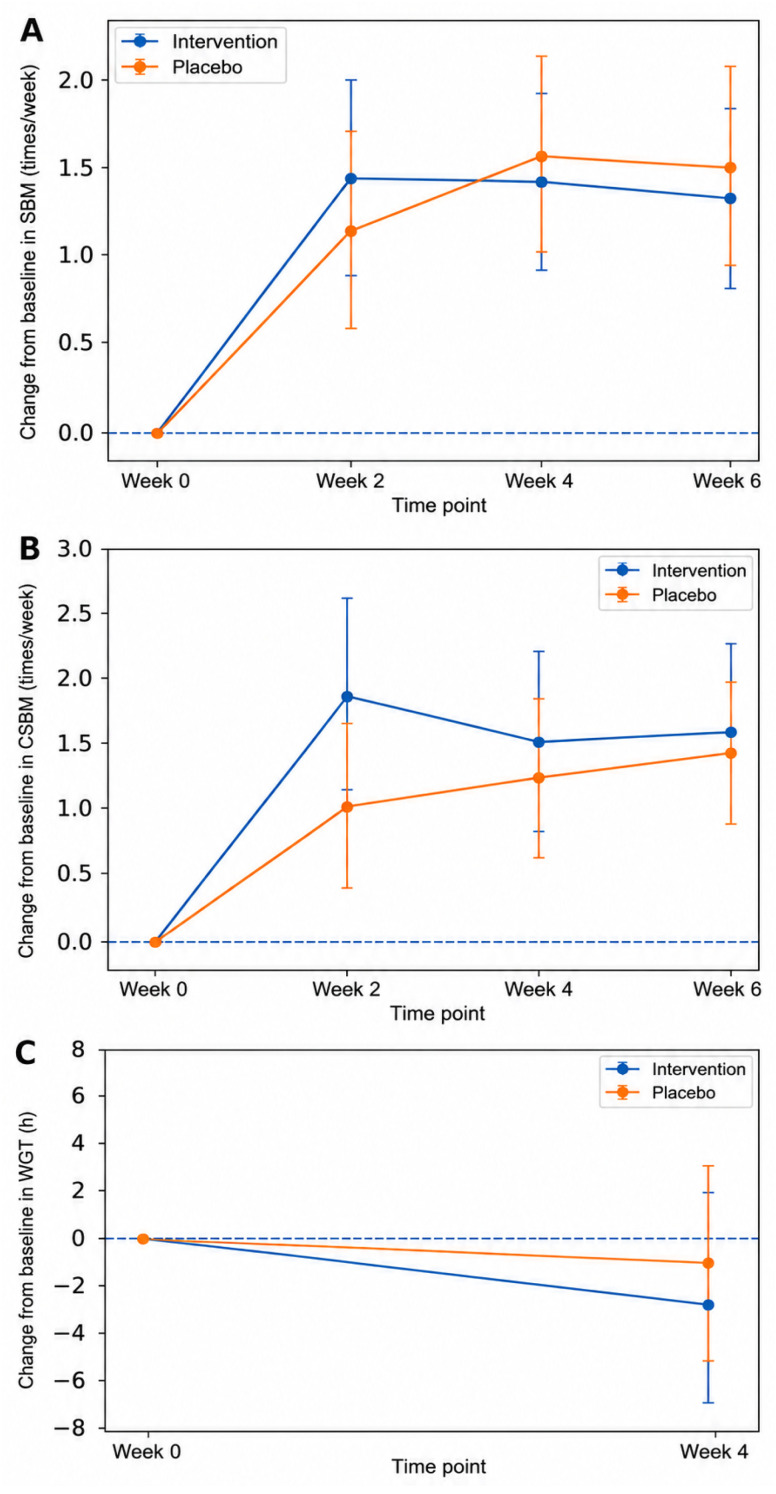
Changes from baseline in bowel movement frequency and whole-gut transit time in the PP population. Changes from baseline are shown for (**A**) SBM, (**B**) CSBM, and (**C**) WGTT. Data are presented as mean changes from baseline with 95% confidence intervals. Week 0 was set as 0 because values represent changes from baseline. WGTT was assessed only at baseline and week 4. Positive and negative values indicate increases and decreases from baseline, respectively. CSBM, complete spontaneous bowel movement; PP, per-protocol; SBM, spontaneous bowel movement; WGTT, whole-gut transit time.

In the PP population, changes from baseline in bowel movement frequency and WGTT are shown in Figure 3 and Table S3. The intervention did not result in significantly greater improvements in SBM, CSBM, or WGTT compared with placebo. At week 4, the between-group difference in SBM change was −0.14 movements/week (95% CI: −0.85 to 0.57; *p* = 0.683), in CSBM change was 0.27 movements/week (95% CI: −0.61 to 1.15; *p* = 0.543), and in WGTT change was −1.55 h (95% CI: −7.65 to 4.55; *p* = 0.614). Similar non-significant between-group differences were observed at weeks 2 and 6 for both SBM and CSBM (Table S3). Both groups showed increased SBM and CSBM frequencies and decreased WGTT over time, with similar trajectories between groups (Figure 3).

ITT analysis of the primary outcome (SBM) and key secondary outcomes (CSBM and WGTT) at week 4 yielded results consistent with the PP analysis, showing no significant between-group differences (Table S4).

#### 3.2.2. Constipation Symptom Severity and Constipation-Related Quality of Life

**Figure 4:**
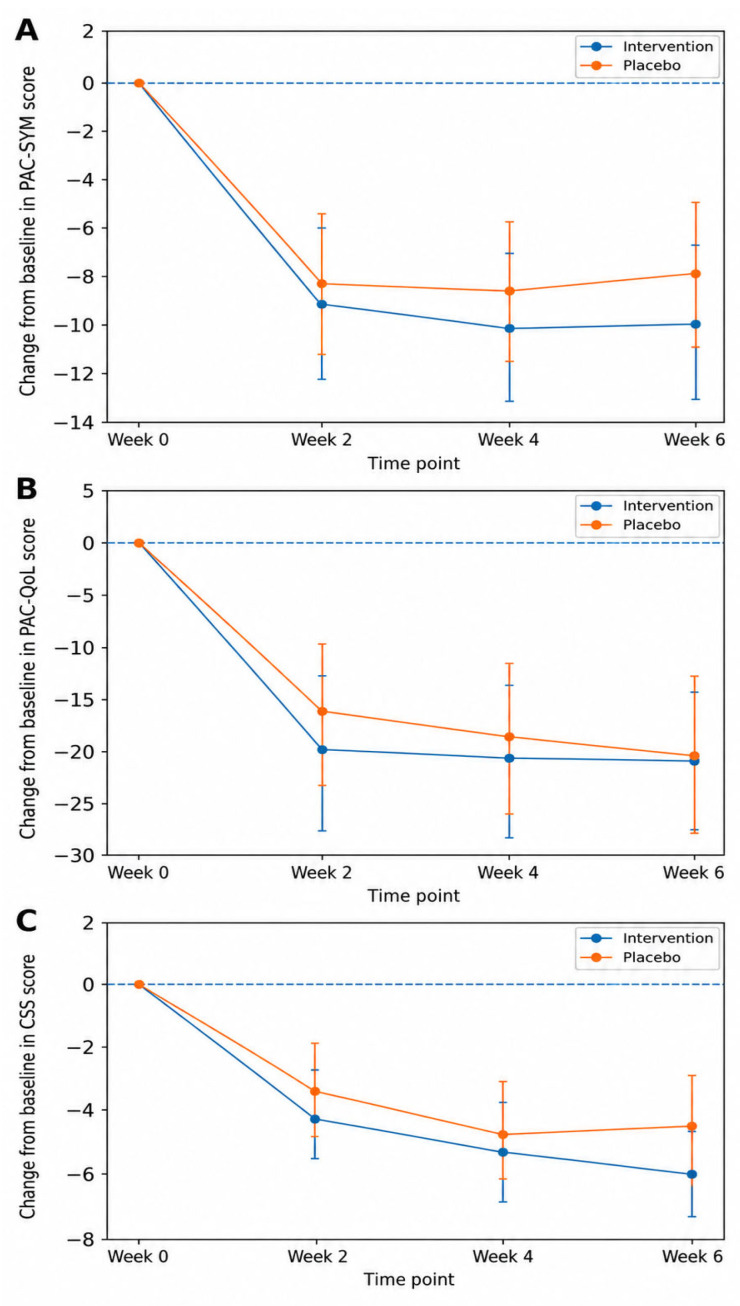
Changes from baseline in constipation symptom severity and constipation-related quality-of-life scores in the PP population. Changes from baseline are shown for (**A**) PAC-SYM, (**B**) PAC-QoL, and (**C**) CSS. Data are presented as mean changes from baseline with 95% confidence intervals. Week 0 was set as 0 because values represent changes from baseline. Negative values indicate improvement. CSS, Constipation Scoring System; PAC-QoL, Patient Assessment of Constipation Quality of Life; PAC-SYM, Patient Assessment of Constipation Symptoms; PP, per-protocol.

In the PP population, changes from baseline in PAC-SYM, PAC-QoL, and CSS scores are shown in Figure 4 and Table S5. Both groups showed reductions in all scores over time, indicating improvements in constipation-related symptoms and quality of life within each group. However, the changes from baseline did not differ significantly between groups at week 4. The between-group differences in change were −1.54 points for PAC-SYM (95% CI: −5.56 to 2.48; *p* = 0.448), −1.95 points for PAC-QoL (95% CI: −11.68 to 7.78; *p* = 0.690), and −0.50 points for CSS (95% CI: −2.18 to 1.18; *p* = 0.556). Similar non-significant between-group differences were observed at weeks 2 and 6 (Table S5). The trajectories of changes were broadly similar between the two groups (Figure 4A–C).

#### 3.2.3. Exploratory Subgroup and Responder Analyses

In the PP population, an exploratory subgroup analysis was performed according to baseline SBM frequency (Table S6). Among participants with baseline SBM ≥ 3 times/week, a greater increase in CSBM was observed in the intervention group than in the placebo group at week 4 (MD = 1.44 movements/week, 95% CI: 0.04 to 2.84; *p* = 0.044). No significant between-group differences were observed for SBM or WGTT in either subgroup, or for CSBM in the baseline SBM < 3 times/week subgroup.

An exploratory responder analysis (≥1 CSBM/week increase from baseline) showed no significant between-group differences in responder rates in the overall population or in either baseline SBM subgroup (Table S7).

### 3.3. Serum Biomarkers

Serum VIP increased significantly from baseline in the intervention group (from 185.57 ± 37.23 to 260.10 ± 32.26 ng/L; *p* < 0.001), whereas it decreased in the placebo group (from 227.16 ± 61.84 to 196.46 ± 56.55 ng/L; *p* < 0.001), yielding a significant between-group difference (MD = 105.23 ng/L, 95% CI: 84.53, 125.93; *p* < 0.001; Table 2). Serum ACh also increased significantly in both groups, with a greater increase in the intervention group (MD = 42.95 ng/L, 95% CI: 3.19, 82.71; *p* = 0.035). Changes in serum 5-HT, NO, and inflammatory cytokines (IL-6, IL-1β, IL-10, TNF-α, and IFN-γ) did not differ significantly between groups (all *p* > 0.05; Table 2).

### 3.4. Adverse Events

No adverse events were reported throughout the study.

## 4. Discussion

In this RCT, we evaluated the effects of a heat-inactivated two-strain *L. paracasei* fermented milk (intervention) on clinical outcomes and serum biomarkers in middle-aged and older adults with FC. The intervention was safe and well tolerated over 4 weeks, but did not significantly improve bowel movement frequency, WGTT, constipation symptom severity, or constipation-related quality of life compared with placebo. Notably, serum VIP and ACh increased significantly in the intervention group, whereas no significant between-group changes were observed in 5-HT, NO, or inflammatory cytokines. These biomarker findings suggest possible involvement of neurotransmitter-related pathways, but should be regarded as exploratory and not as evidence of a direct mechanism or clinical efficacy.

Although meta-analyses have shown that some probiotic or postbiotic interventions may increase bowel movement frequency, shorten intestinal transit time, or improve stool consistency [26,43], the evidence is characterized by substantial heterogeneity across strains, populations, and study designs [25,44]. Therefore, the lack of significant between-group clinical benefit in the present study should be interpreted cautiously in the context of the substantial heterogeneity of microbiota-targeted interventions. Similar findings have been reported in previous microbiota-targeted intervention studies in FC. Mazlyn et al. reported that although constipation severity scores and bowel movement frequency improved from baseline after *Lactobacillus casei* Shirota intervention, the differences compared with placebo did not reach statistical significance [22]. The substantial within-group improvements observed in both groups in the present study are consistent with the well-documented placebo response in FC trials. A systematic review by Chen et al. reported that placebo response rates in chronic constipation trials range from 20% to 40% across different clinical outcomes, which may be related to increased attention to bowel habits, regular diary recording, behavioral modification, and regression to the mean [45]. Another important consideration is that the comparator in this trial should not be considered biologically inert. Although it did not contain the study strains, it shared the same milk-based matrix and several functional ingredients with the intervention product, including fermentable fibers such as polydextrose, soluble soybean polysaccharides, and pectin. These components may influence stool bulk, intestinal fermentation, and bowel function, while dairy-derived nutrients and peptides may also have physiological activity [46,47].

Several factors specific to the present trial may have further limited the detection of between-group differences. First, baseline SBM frequency was approximately 3 movements per week, close to the Rome IV diagnostic threshold for FC [1], indicating relatively mild impairment in bowel frequency in the enrolled population. Participants with near-normal baseline values may have limited room for improvement, potentially limiting the detectable between-group difference. Second, the 4-week intervention may have been too short for postbiotic-mediated effects to produce measurable clinical changes, given that heat-inactivated postbiotics act through retained structural components and metabolites rather than through colonization [24,25]; the time course over which these mechanisms translate into improved bowel function remains unclear. Third, the sample size calculation was based on an expected between-group difference of 0.98 SBM/week derived from earlier probiotic trials [43]. The observed difference of −0.14 SBM/week suggests that this effect size may have been overestimated, highlighting the need for more conservative estimates in future postbiotic trials.

A notable exploratory finding of this study was the greater increase in serum ACh and VIP in the intervention group compared with placebo. ACh and VIP are important signaling molecules involved in enteric nervous system regulation. ACh is a major excitatory neurotransmitter that promotes smooth muscle contraction and propulsive motility, whereas VIP is an important inhibitory neurotransmitter involved in smooth muscle relaxation, intestinal secretion, and sphincter regulation [48,49]. Normal intestinal propulsion requires coordinated activation of excitatory and inhibitory neural pathways, allowing contraction and relaxation to occur in an organized manner along the direction of propulsion. Therefore, concurrent increases in ACh and VIP are not necessarily opposing biological responses, but may be compatible with modulation of neuroregulatory pathways related to intestinal motility. Previous animal and clinical studies have reported alterations in ACh- and VIP-related signaling in constipation models and in patients with slow-transit constipation [50,51,52].

However, these biomarker findings should be interpreted cautiously. This study measured circulating serum ACh and VIP rather than local neurotransmitter activity in the intestinal mucosa, enteric nervous system, or colonic tissue. Therefore, the results do not provide direct evidence that the intervention corrected a local neurotransmitter abnormality or improved enteric neural function. The potential mechanisms underlying the observed biomarker changes remain unclear. Retained microbial structural components of heat-inactivated bacteria, such as peptidoglycan, lipoteichoic acid, and cell wall polysaccharides, may interact with intestinal epithelial, immune, enteroendocrine, or neural-associated pathways [53,54]. In addition, because both *L. paracasei* strains were used to ferment the milk before heat inactivation, the final product may also contain strain- and process-dependent fermentation-derived metabolites, including organic acids, free amino acids, bioactive peptides, fatty acid-related metabolites, γ-aminobutyric acid, and exopolysaccharide-like compounds [24,55,56]. These metabolites may contribute to the biological properties of fermented dairy products and postbiotic preparations, but their presence and concentrations were not directly quantified in the present study. The absence of significant between-group changes in serum 5-HT, NO, and inflammatory cytokines does not support broad neuroimmune or inflammatory activation.

Importantly, the increases in serum ACh and VIP were not accompanied by significant between-group improvements in SBM, CSBM, WGTT, or constipation-related symptom scores. This dissociation between biomarker changes and clinical outcomes suggests that the observed changes may have been insufficient in magnitude or duration to produce measurable improvements in defecatory function, or that circulating biomarker levels may not accurately reflect functionally relevant local changes in the intestine. Functional constipation is a multifactorial disorder involving not only neurotransmitter regulation but also pelvic floor function, dietary factors, psychological comorbidities, visceral sensitivity, and gut luminal or microbial factors [37]. Thus, changes in selected circulating neurotransmitter-related biomarkers alone may be insufficient to overcome these other contributors. Overall, the serum ACh and VIP results should be regarded as hypothesis-generating biomarker findings that suggest possible involvement of neurotransmitter-related pathways, rather than evidence of a direct mechanism or clinical efficacy. Further studies incorporating local intestinal, fecal, or microbiota-related mechanistic readouts are needed to clarify the functional relevance of these circulating biomarker changes.

This study has several limitations. First, it was conducted at a single center in Beijing, and the participants were predominantly women (80%), which may have limited generalizability to other populations. Second, the 4-week intervention duration may have been too short to capture the full trajectory of clinical response to a postbiotic intervention. Third, habitual dietary intake—particularly dietary fiber—was not rigorously controlled, and variations in fiber consumption may have confounded bowel function outcomes. Fourth, the divergent changes in serum VIP between groups—an increase in the intervention group and an unexpected decrease in the placebo group—may reflect regression to the mean, seasonal variation, or unmeasured confounders; this bidirectional pattern warrants cautious interpretation of the between-group difference and highlights the need for multiple baseline measurements in future studies. Fifth, we did not perform targeted or untargeted metabolomic, peptidomic, or exopolysaccharide analyses of the fermented milk product. Therefore, the specific fermentation-derived metabolites produced by the two *L. paracasei* strains could not be identified or quantified, and their contribution to the observed serum ACh and VIP changes remains speculative. Sixth, the baseline SBM-stratified analysis and responder analysis were exploratory and were not powered for subgroup comparisons; no adjustment for multiple comparisons was performed. Among participants with baseline SBM ≥ 3 times/week, a greater CSBM improvement was observed in the intervention group, but this finding should be interpreted with caution due to the small subgroup sample sizes and the lack of adjustment for multiple comparisons, and requires confirmation in adequately powered future studies.

## 5. Conclusions

In this randomized trial, 4 weeks of intervention with a heat-inactivated two-strain *L. paracasei* fermented milk was well tolerated but did not significantly improve bowel movement frequency, whole-gut transit time, constipation-related symptoms, or quality of life compared with placebo in middle-aged and older adults with FC. Although serum ACh and VIP increased significantly in the intervention group, these biomarker changes were not accompanied by significant clinical improvements within the intervention period and should therefore be regarded as exploratory. Future studies with longer intervention durations, adequate dietary control, assessment of fecal microbiota and local intestinal function, and designs that better distinguish shared matrix effects from postbiotic-specific effects are warranted.

## Figures and Tables

**Table 1:** Baseline characteristics of participants (ITT population).

Indicator	Intervention	Placebo	*p* Value
	*n* = 50	*n* = 50	
Men, *n* (%)	10 (20.0%)	10 (20.0%)	1.000
Women, *n* (%)	40 (80.0%)	40 (80.0%)	
Age, years	63.00 ± 7.13	60.74 ± 8.14	0.143
BMI, kg/m^2^	24.02 ± 2.65	24.39 ± 2.81	0.497
SBM, times/week	2.94 ± 1.27	2.76 ± 1.19	0.466
CSBM, times/week	2.00 ± 1.18	2.14 ± 1.43	0.594
WGTT, h	38.37 ± 17.31	35.43 ± 15.52	0.374

Data are expressed as mean ± SD or *n*. The *p* values were calculated using unpaired t-tests for continuous variables and chi-square tests for categorical variables. BMI, body mass index; CSBM, complete spontaneous bowel movement; SBM, spontaneous bowel movement; WGTT, whole-gut transit time.

**Table 2:** Between-group comparison of changes in serum neurotransmitter-related biomarkers and inflammatory cytokines (PP population).

Outcome	Group	Baseline	Week 4	Change from Baseline, Δ	Mean Difference (95% CI), *p* Value
5-HT, ng/L	Intervention	470.65 ± 58.68	329.83 ± 58.25 ***	−148.54 ± 97.66	−11.09 (−71.45, 49.27), *p* = 0.715
	Placebo	466.33 ± 182.32	328.88 ± 78.22 ***	−137.45 ± 185.61	
ACh, ng/L	Intervention	230.65 ± 41.50	358.44 ± 45.47 ***	127.79 ± 54.54	42.95 (3.19, 82.71), *p* = 0.035
	Placebo	279.86 ± 134.79	364.70 ± 94.14 ***	84.84 ± 126.64	
VIP, ng/L	Intervention	185.57 ± 37.23	260.10 ± 32.26 ***	74.53 ± 50.69	105.23 (84.53, 125.93) *p* < 0.001
	Placebo	227.16 ± 61.84	196.46 ± 56.55 ***	−30.70 ± 51.44	
NO, µmol/mL	Intervention	17.19 ± 6.52	17.93 ± 6.73	0.73 ± 5.29	0.85 (−1.67, 3.37), *p* = 0.502
	Placebo	19.11 ± 6.42	18.99 ± 6.34	−0.12 ± 7.02	
IL-6, ng/L	Intervention	23.42 ± 2.80	18.12 ± 3.39 ***	−5.30 ± 4.23	0.59 (−0.96, 2.14), *p* = 0.450
	Placebo	23.77 ± 2.57	17.88 ± 2.87 ***	−5.89 ± 3.36	
IL-1β, pg/mL	Intervention	46.29 ± 6.03	31.10 ± 4.97 ***	−15.18 ± 6.40	−1.09 (−4.21, 2.03), *p* = 0.485
	Placebo	45.53 ± 5.83	31.44 ± 5.50 ***	−14.09 ± 8.77	
IL-10, ng/L	Intervention	330.17 ± 53.61	484.12 ± 56.00 ***	153.95 ± 73.82	−1.29 (−31.91, 29.33), *p* = 0.933
	Placebo	323.07 ± 53.77	478.31 ± 52.91 ***	155.24 ± 77.24	
TNF-α, ng/L	Intervention	499.58 ± 54.64	375.76 ± 46.14 ***	−123.82 ± 66.73	20.96 (−7.74, 49.66), *p* = 0.150
	Placebo	504.86 ± 55.29	360.08 ± 45.18 ***	−144.78 ± 74.64	
IFN-γ, ng/L	Intervention	488.55 ± 58.37	365.69 ± 55.17 ***	−122.86 ± 80.69	−20.84 (−57.01, 15.33), *p* = 0.255
	Placebo	473.11 ± 58.27	371.10 ± 61.58 ***	−102.02 ± 96.96	

Data are presented as mean ± SD. Δ represents the change from baseline to Week 4. Mean differences and 95% CIs were calculated as ΔIntervention − ΔPlacebo. *p* values for between-group comparisons were derived from independent t-tests. Asterisks indicate significant within-group differences compared with baseline (paired t-tests): *** *p* < 0.001. 5-HT, 5-hydroxytryptamine (serotonin); ACh, acetylcholine; IFN-γ, interferon-gamma; IL, interleukin; NO, nitric oxide; TNF-α, tumor necrosis factor-alpha; VIP, vasoactive intestinal peptide.

## Data Availability

Dataset available on request from the authors. The raw data supporting the conclusions of this article will be made available by the authors on request.

## Supplementary Materials

The following supporting information can be downloaded at: https://www.mdpi.com/article/10.3390/nu18132114/s1, Table S1: Study’s inclusion and exclusion criteria; Table S2: Composition and nutritional information of the intervention and placebo products; Table S3: Between-group comparison of changes in bowel movement frequency and whole-gut transit time (PP population); Table S4: Intention-to-treat analysis of primary and key secondary outcomes at week 4; Table S5: Between-group comparison of changes in constipation symptom severity and constipation-related quality-of-life scores (PP population); Table S6: Exploratory subgroup analysis of changes in bowel movement frequency and whole-gut transit time at week 4, stratified by baseline SBM frequency (PP population); Table S7: Exploratory responder analysis in the PP population.

## Abbreviations

The following abbreviations are used in this manuscript:

5-HT	5-Hydroxytryptamine
ACh	Acetylcholine
AE	Adverse event
BMI	Body mass index
CFU	Colony-forming units
CI	Confidence interval
CSBM	Complete spontaneous bowel movement
CSS	Constipation Scoring System
CV	Coefficient of variation
ELISA	Enzyme-linked immunosorbent assay
ENS	Enteric nervous system
FC	Functional constipation
IFN-γ	Interferon-γ
IL-10	Interleukin-10
IL-1β	Interleukin-1β
IL-6	Interleukin-6
IQR	Interquartile range
MD	Mean difference
NO	Nitric oxide
PAC-QoL	Patient Assessment of Constipation Quality of Life
PAC-SYM	Patient Assessment of Constipation Symptoms
PI	Principal investigator
PPS	Per-protocol set
RCT	Randomized controlled trial
SAE	Serious adverse event
SBM	Spontaneous bowel movement
SD	Standard deviation
TNF-α	Tumor necrosis factor-α
VIP	Vasoactive intestinal peptide
WGTT	Whole-gut transit time
