# Supplementary material for: A Heat-Inactivated Two-Strain Lacticaseibacillus paracasei Fermented Milk as a Postbiotic for Functional Constipation: A Randomized, Double-Blind, Placebo-Controlled Trial
Source: Nutrients. 2026 Jun 28;18(13):2114. doi: 10.3390/nu18132114 (PMC13363550; doi:10.3390/nu18132114)
Supplement: Supplementary file 1 [file nutrients-18-02114-s001.zip › nutrients-4372937-supplementary.pdf]

Supplementary Materials:

Recipe for blue-dyed cupcakes

The blue-dyed cupcakes used for whole-gut transit time assessment were prepared using 245 g all-purpose flour, 15 g baking powder, 200 g granulated sugar, 100 g vegetable oil, 230 g water, 1 teaspoon vanilla extract, and 6 g Wilton blue food coloring. This recipe yielded approximately 12 blue-dyed cupcakes.

Table S1. Study's inclusion and exclusion criteria

Inclusion criteria
1) Age: 45-75 years old.
2) BMI：18.5-34.9 Kg/m2
3) Meets the Rome IV diagnostic criteria for constipation;<br>①Must include 2 or more of the following:<br>a. More than 1/4 (25%) of bowel movements are dry balls or hard stools (Bristol Stool Form Scale 1-2)<br>b. More than 1/4 (25%) of cases have incomplete bowel movements<br>c. More than 1/4 (25%) of bowel movements have anorectal obstruction/blockage sensation<br>d. More than 1/4 (25%) of bowel movements require manual assistance (such as finger assisted defecation, pelvic floor support)<br>e. Spontaneous bowel movements (SBM) less than 3 times per week<br>②When not using laxatives, there is rarely loose stool<br>③The symptoms before diagnosis have been present for at least 6 months and have met the above diagnostic criteria in the past 3 months.
4) Voluntarily participate in this experiment and sign the informed consent form.
**Exclusion criteria**
1) Have serious gastrointestinal diseases (gastric or duodenal ulcers, acute enteritis, ulcerative colitis) or significant organic lesions (colon cancer, tumors, etc.).
2) Suffering from type 1 diabetes, Parkinson's disease or any disease requiring chronic treatment.
3) Two weeks prior to the experiment, use H2 receptor antagonists, proton pump inhibitors (CPPI), or consume probiotic or prebiotic preparations.
4) Use any of the following medications three days before randomization:<br>a.Prokinetic agents (Tegaserod, Domperidone, Cisapride, Metoclopramide, Erythromycin)<br>b. Containing opiate drugs<br>c. Antispasmodic agents (atropine, hyoscyamine, scopolamine, glycopyrrolate)<br>d. Chronic nonsteroidal anti-inflammatory drugs (NSAIDs)<br>e. Antibiotics and laxatives.
5) Excessive smoking and drinking.
6) Women who are pregnant, breastfeeding, or planning to become pregnant within the next 6 months.
7) Allergic to milk and soybeans.

Table S2. Composition and nutritional information of the intervention and placebo products

Ingredient	Intervention (Changyi fermented milk)	Placebo
Drinking water	✓	✓
High fructose corn syrup	✓	✓
Milk powder (≥ 2.4%)	✓	✓
Polydextrose	✓	✓
Glucose	✓	✓
Lacticaseibacillus paracasei (strain K56; heat-inactivated)	✓	✗
Lacticaseibacillus paracasei (strain G078; heat-inactivated)	✓	✗
Soluble soybean polysaccharides	✓	✓
Pectin	✓	✓
Citric acid	✓	✓
Sucralose	✓	✓
Acesulfame potassium	✓	✓
Food-grade flavoring	✓	✓
Nutritional composition		
Energy (kJ/100 mL)	193	193
Protein (g/100 mL)	0.7	0.7
Fat (g/100 mL)	0	0
Carbohydrate (g/100 mL)	9.4	9.4
Dietary fiber (as polydextrose)	0.7	0.7
Na (mg/100 mL)	75	75
Lacticaseibacillus paracasei K56 and G078 (CFU equivalents per 100 mL prior to inactivation)	$3.9\times 10^{\wedge 10}$	0

Table S3. Between-group comparison of changes in bowel movement frequency and whole-gut transit time (PP population)

Outcome	Time point	Intervention	Placebo	Δ Intervention	Δ Placebo	MD (95% CI), *P* value
SBM, times/week	Week 0	3.00 ± 1.25	2.77 ± 1.21	—	—	—
	Week 2	4.46 ± 2.11***	3.94 ± 1.98***	1.46 ± 1.86	1.17 ± 1.91	0.29 (-0.47, 1.05), *P* = 0.449
	Week 4	4.44 ± 2.07***	4.35 ± 1.94***	1.44 ± 1.65	1.58 ± 1.83	-0.14 (-0.85, 0.57), *P* = 0.683
	Week 6	4.35 ± 2.15***	4.29 ± 2.00***	1.35 ± 1.73	1.52 ± 1.88	-0.17 (-0.90, 0.56), *P* = 0.652
CSBM, times/week	Week 0	2.04 ± 1.18	2.15 ± 1.44	—	—	—
	Week 2	3.92 ± 2.35***	3.21 ± 2.24***	1.88 ± 2.42	1.06 ± 2.12	0.82 (-0.10, 1.74), *P* = 0.083

	Week 4	3.58 ± 2.27***	3.42 ± 2.14***	1.54 ± 2.29	1.27 ± 2.05	0.27 (-0.61, 1.15), *P* = 0.543
	Week 6	3.65 ± 2.39***	3.60 ± 1.94***	1.60 ± 2.32	1.45 ± 1.82	0.14 (-0.70, 0.98), *P* = 0.733
WGTT, h	Week 0	38.85 ± 17.50	35.72 ± 15.78	—	—	—
	Week 4	36.48 ± 18.21	34.91 ± 17.73	−2.37 ± 15.80	−0.82 ± 14.25	-1.55 (-7.65, 4.55), *P* = 0.614

Data are presented as mean ± SD. Δ represents the change from baseline to each corresponding time point. WGTT was assessed only at baseline and week 4. Mean difference and 95% confidence intervals (CIs) were calculated as Δ Intervention − Δ Placebo. *P* values for between-group comparisons were derived from independent t-tests. Asterisks indicate significant within-group differences compared with baseline (paired t-tests): *** *P* < 0.001. CSBM, complete spontaneous bowel movement; SBM, spontaneous bowel movement; WGTT, whole-gut transit time.

Table S4. Intention-to-treat analysis of primary and key secondary outcomes at week 4

**Outcome**	**Group**	**Baseline**	**Week 4**	**Change from baseline, Δ**	**Mean Difference (95% CI), *P* value**
SBM, times/week	Intervention	2.94 ± 1.27	4.38 ± 2.05***	1.44 ± 1.62	−0.16 (−0.84, 0.52), *P* = 0.641
	Placebo	2.76 ± 1.19	4.36 ± 1.90***	1.60 ± 1.80	
CSBM, times/week	Intervention	2.00 ± 1.18	3.56 ± 2.22***	1.56 ± 2.24	0.26 (−0.59, 1.11), *P* = 0.544
	Placebo	2.14 ± 1.43	3.44 ± 2.10***	1.30 ± 2.02	
WGTT, h	Intervention	38.37 ± 17.32	36.04 ± 17.97	−2.33 ± 15.48	−1.52 (−7.37, 4.32), *P* = 0.606
	Placebo	35.43 ± 15.52	34.63 ± 17.42	−0.80 ± 13.96	

Data are presented as mean ± SD. Δ represents the change from baseline to week 4. Mean difference and 95% confidence intervals (CIs) were calculated as Δ Intervention − Δ Placebo. *P* values for between-group comparisons were derived from independent t-tests. Asterisks indicate significant within-group differences compared with baseline (paired t-tests): *** *P* < 0.001. CSBM, complete spontaneous bowel movement; SBM, spontaneous bowel movement; WGTT, whole-gut transit time.
Missing data for the 4 participants who withdrew were imputed using last observation carried forward (LOCF).

Table S5. Between-group comparison of changes in constipation symptom severity and constipation-related quality-of-life scores (PP population)

Outcome	Time point	Intervention	Placebo	Δ Intervention	Δ Placebo	MD (95% CI),
PAC-SYM, score	Week 0	19.81 ± 9.18	18.27 ± 9.34	—	—	—
	Week 2	10.60 ± 7.82***	9.83 ± 7.48***	-9.21 ± 10.65	-8.44 ± 9.75	-0.77 (-4.91, 3.37),
	Week 4	9.58 ± 6.83***	9.58 ± 8.01***	-10.23 ± 10.23	-8.69 ± 9.61	-1.54 (-5.56, 2.48),
	Week 6	9.79 ± 8.18***	10.27 ± 7.36***	-10.02 ± 10.77	-8.00 ± 9.93	-2.02 (-6.22, 2.18),
PAC-QoL, score	Week 0	49.40 ± 21.77	45.67 ± 23.30	—	—	—
	Week 2	28.88 ± 19.02***	28.73 ± 17.64***	-20.52 ± 24.94	-16.94 ± 23.32	-3.58 (-13.37, 6.21),
	Week 4	28.06 ± 18.77***	26.29 ± 17.39***	-21.33 ± 24.25	-19.38 ± 23.77	-1.95 (-11.68, 7.78),
	Week 6	27.92 ± 19.49***	24.69 ± 17.33***	-21.48 ± 21.75	-20.98 ± 24.15	-0.50 (-9.82, 8.82),
CSS, score	Week 0	12.96 ± 3.56	12.29 ± 3.70	—	—	—
	Week 2	8.94 ± 3.76***	8.98 ± 3.78***	-4.02 ± 3.97	-3.31 ± 4.04	-0.71 (-2.33, 0.91),
	Week 4	7.92 ± 4.16***	7.75 ± 3.15***	-5.04 ± 4.36	-4.54 ± 3.91	-0.50 (-2.18, 1.18),
	Week 6	7.42 ± 3.48***	7.92 ± 3.29***	-5.54 ± 3.71	-4.32 ± 4.18	-1.22 (-2.82, 0.38),

Data are presented as mean ± SD. Δ represents the change from baseline to each corresponding time point. Mean differences and 95% CIs were calculated as ΔIntervention − ΔPlacebo. *P* values for between-group comparisons were derived from independent t-tests. Asterisks indicate significant within-group differences compared with baseline (paired t-tests): *** $P < 0.001$. CSS, Constipation Scoring System; PAC-SYM, Patient Assessment of Constipation Symptoms; PAC-QoL, Patient Assessment of Constipation Quality of Life.

Table S6. Exploratory subgroup analysis of changes in bowel movement frequency and whole-gut transit time at week 4, stratified by baseline SBM frequency (PP population)

Outcome	Baseline SBM subgroup	Group	Δ Week 4	MD (95% CI)	*P* value
SBM, times/week	< 3 times/week	Intervention	1.56 ± 1.70	-0.06 (-0.94, 0.82)	0.894
		Placebo	1.62 ± 1.96		
	≥ 3 times/week	Intervention	1.19 ± 1.56	-0.26 (-1.43, 0.91)	0.642
		Placebo	1.45 ± 1.37		
CSBM, times/week	< 3 times/week	Intervention	1.09 ± 2.23	-0.07 (-1.13, 0.99)	0.898
		Placebo	1.16 ± 2.18		
	≥ 3 times/week	Intervention	2.84 ± 2.09	1.44 (0.04, 2.84)	0.044
		Placebo	1.40 ± 1.43		
WGTT, h	< 3 times/week	Intervention	-2.04 ± 17.32	-1.11 (-8.92, 6.70)	0.778
		Placebo	-0.93 ± 14.73		
	≥ 3 times/week	Intervention	-3.04 ± 12.70	-2.62 (-13.18, 7.94)	0.613
		Placebo	-0.42 ± 13.15		

Data are presented as mean ± SD. Δ represents the change from baseline to week 4. Mean difference and 95% confidence intervals (CIs) were calculated as Δ Intervention − Δ Placebo. *P* values for between-group comparisons were derived from independent t-tests. Sample sizes are shown as intervention/placebo: baseline SBM <3 times/week, n = 32/37; baseline SBM ≥3 times/week, n = 16/11. This analysis was exploratory and was not adjusted for multiple comparisons. CSBM, complete spontaneous bowel movement; PP, per-protocol; SBM, spontaneous bowel movement; WGTT, whole-gut transit time.

Table S7. Exploratory responder analysis in the PP population

Baseline SBM subgroup	Intervention group, n/N (%)	Placebo group, n/N (%)	Difference, percentage points	*P* value
Overall population	33/48 (68.8%)	35/48 (72.9%)	-4.2	0.662
SBM <3 times/week	19/32 (59.4%)	27/37 (73.0%)	-13.6	0.263
SBM ≥3 times/week	14/16 (87.5%)	8/11 (72.7%)	+14.8	0.331

Responders were defined as participants who achieved an increase of at least one CSBM per week from baseline. Difference was calculated as the responder rate in the intervention group minus that in the placebo group. *P* values were calculated using Fisher's exact test to account for small expected frequencies in some subgroups. This analysis was exploratory and was not adjusted for multiple comparisons. CSBM, complete spontaneous bowel movement; PP, per-protocol; SBM, spontaneous bowel movement.
